# Sound Frequency and Aural Selectivity in Sound-Contingent Visual Motion Aftereffect

**DOI:** 10.1371/journal.pone.0036803

**Published:** 2012-05-23

**Authors:** Maori Kobayashi, Wataru Teramoto, Souta Hidaka, Yoichi Sugita

**Affiliations:** 1 Research Institute of Electrical Communication, Tohoku University, Sendai, Miyagi, Japan; 2 Department of Psychology, Graduate School of Arts and Letters, Tohoku University, Sendai, Miyagi, Japan; 3 Department of Psychology, Rikkyo University, Niiza, Saitama, Japan; 4 Neuroscience Research Institute, National Institute of Advanced Industrial Science and Technology (AIST), Tsukuba, Ibaraki, Japan; Nothwestern University, United States of America

## Abstract

**Background:**

One possible strategy to evaluate whether signals in different modalities originate from a common external event or object is to form associations between inputs from different senses. This strategy would be quite effective because signals in different modalities from a common external event would then be aligned spatially and temporally. Indeed, it has been demonstrated that after adaptation to visual apparent motion paired with alternating auditory tones, the tones begin to trigger illusory motion perception to a static visual stimulus, where the perceived direction of visual lateral motion depends on the order in which the tones are replayed. The mechanisms underlying this phenomenon remain unclear. One important approach to understanding the mechanisms is to examine whether the effect has some selectivity in auditory processing. However, it has not yet been determined whether this aftereffect can be transferred across sound frequencies and between ears.

**Methodology/Principal Findings:**

Two circles placed side by side were presented in alternation, producing apparent motion perception, and each onset was accompanied by a tone burst of a specific and unique frequency. After exposure to this visual apparent motion with tones for a few minutes, the tones became drivers for illusory motion perception. However, the aftereffect was observed only when the adapter and test tones were presented at the same frequency and to the same ear.

**Conclusions/Significance:**

These findings suggest that the auditory processing underlying the establishment of novel audiovisual associations is selective, potentially but not necessarily indicating that this processing occurs at an early stage.

## Introduction

**Figure 1:**
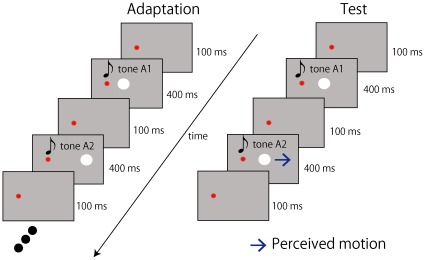
Experimental design. In an adaptation period, observers were exposed to apparent visual motion for 18 min, during which two white circles placed side by side were presented in alternation. The onset of the left circle was accompanied by tone A1 and the right circle by tone A2. In the test phase, a white circle was presented twice. The circle was perceived as lateral motion in the same direction as the previously presented apparent motion when the onset of the circle was synchronized to tones of alternating frequencies. When the onset of the first circle was synchronized with tone A1 and the second circle with tone A2, the circle appeared to move from left to right. The strength of this illusory motion was quantified by a motion-nulling procedure.

Integration of information received via multiple senses is thought to be fundamental to perception and cognition. The combination of inputs from different senses can function to reduce perceptual ambiguity [1]–[4] and enhance stimulus detection [5]–[7]. However, the brain has to evaluate whether signals in different modalities come from a common external event or object. One possible strategy for this task is to form associations between the inputs received via different senses. This strategy would be rather effective because signals in different modalities from a common external event would then be aligned spatially and temporally. Indeed, our previous studies demonstrated that a strong association between a sound sequence and visual motion is easily formed within a short period and that, after forming the association, sounds can trigger visual motion perception for a static visual object [8]. In an adaptation period, two circles placed side by side were presented in alternation, and each onset was accompanied by a tone burst of a specific and unique frequency. After a few minutes of such exposure to this visual apparent motion accompanied by the tones, the tones began to trigger illusory visual motion in response to a static visual stimulus (Figure 1).

Such a sound-contingent visual aftereffect might be mediated in the higher cortical areas, where cells receive both visual and auditory inputs and have large receptive fields that provide neural substrates for translational invariance. If this were the case, an aftereffect would be observed even in retinal positions other than those exposed. However, surprisingly, the sound-contingent visual aftereffect was observed only at the retinal position previously exposed to an apparent motion with tone bursts [8]. This finding indicates that visual processing units that have selectivity regarding the retinal position are responsible for the aftereffect. If such selectivity could also be observed in the auditory domain, we could consider that the sound-contingent visual aftereffect involves relatively early sensory processing of each modality. In order to clarify the auditory nature of the aftereffect, we examined the frequency selectivity and the ear’s selectivity in the sound-contingent visual motion aftereffect.

## Materials and Methods

### Ethics Statement

Informed written consent was obtained from each participant before the experiments were conducted. All procedures were approved by the ethics committee of the Research Institute of Electrical Communication of Tohoku University.

### Participants

There were eight participants, including the authors, all of whom had normal hearing and normal or corrected-to-normal vision. Except for the authors, the participants were naïve to the purpose of the experiments.

### Stimuli and Apparatus

The visual stimuli were presented on a 24-inch CRT display (refresh rate: 60 Hz) at a viewing distance of 1 m. A red circle (0.4° in diameter; 17.47 cd/m^2^) for fixation and two white circles (5.12 cd/m^2^, 1.0° in diameter) were presented on a black background. The auditory stimuli were tone bursts (sampling frequency 44.1 kHz, 85 dB SPL, 50 ms in duration with a 5-ms rise-and-fall time) transmitted to both ears through headphones. The range of sound frequency was from 200 Hz to 4200 Hz (the details provided in the next section). Using a digital oscilloscope, we confirmed that the onset of the visual and the auditory stimuli was synchronized. The participants were instructed to place their heads on a chin rest. All experiments were conducted in a dark room.

### Procedures

#### Experiment 1: Frequency selectivity experiment

The strength of the illusory motion was measured before and after exposure to visual apparent motion with tone bursts (pre- and post-exposure sessions, respectively).

In the exposure phase, two white circles were placed side by side and presented in alternation. The distance between the two circles was 5.0°. The center between the two circles was 10.0° right of fixation. The duration of the presentation of each circle was 400 ms, with a stimulus onset asynchrony of 500 ms. For half of the participants, the onset of the leftward circle was synchronized with a tone burst of high (2100 Hz) frequency and the rightward circle with a tone burst of low (400 Hz) frequency. For the other half, the relationship was reversed. Participants were asked to keep looking at the fixation and were exposed to the apparent visual motion accompanied by tone bursts for 18 min.

In the pre- and post-exposure sessions, the strength of the illusory motion was quantified by a motion-nulling procedure (e.g., [9]). The degree of visual displacement that corresponded to a point of subjective stationarity (PSS) was measured using the method of constant stimuli. The order of the session was randomized and counterbalanced among the participants, and in the second session, a 3- min re-adaptation period was introduced. In each session, two circles (each with a duration of 400 ms) were presented with 500 ms of a stimulus onset asynchrony (SOA), synchronized with two tone bursts. In the rightward sound condition, the first visual stimulus was synchronized with the tone that accompanied the leftward circle during the exposure to apparent motion, and the second stimulus was synchronized with the tone that accompanied the rightward circle. In the leftward sound condition, the relationship was reversed. The no-sound condition was also included. The visual stimulus was displaced 0.12°, 0.24°, 0.48°, or 0.96° from left to right, or vice versa. The degree of displacement and the sound condition were randomized from trial to trial. The participants were asked to judge whether the visual stimulus moved leftward or rightward. In order to examine the sound frequency selectivity, we set seven pairs of test sound frequencies: 200–1050, 283–1485, 336–1766, 400–2100, 476–2497, 566–2970, and 800–4200 Hz. These frequencies were different from the adaptation frequencies by −1, −1/2, −1/4, 0, 1/4, 1/2, and 1 octave. Twenty trials were conducted for each pair of sound frequencies. The order was reversed for the other half.

In order to confirm the results of our generalization tests, another set of experiments using the same procedure was conducted using an exposure frequency pair of 566 and 2970 Hz. For these experiments, we also set the following seven pairs of test sound frequencies: 283–1485, 400–2100, 476–2496, 566–2970, 673–3532, 800–4200, and 1132–5940. These frequencies were different from the adaptation frequencies by −1, −1/2, −1/4, 0, 1/4, 1/2, and 1 octave. Previous studies on frequency selectivity showed that there is no interference between 400 and 2100 Hz, and between 566 and 2970 Hz [10]. These additional experiments were performed for the left visual field in order to prevent the possible influences of the aftereffect by the 400–2100 adapter–because it had already been shown that sound-contingent visual motion aftereffects have rigid localization [8]–so that the effect could be observed only at the retinal position previously exposed to apparent motion with tone bursts.

#### Experiment 2: Ear selectivity experiment

The same methods were employed as in Experiment 1, except for the following. The frequencies of the tone bursts were 1000 and 1500 Hz. These frequencies were selected in order to avoid the influences of Experiment 1; we found that the sound contingent visual motion aftereffect lasts for a few days [8]. Since some participants remained the same between the two experiments, we changed the frequencies of sounds in Experiment 2. Further, it could be assumed that there was little interference between two frequencies from the results showing sharp sound frequency selectivity of the aftereffect in Experiment 1. They were presented monaurally. The tones were presented to the left ear of the participants (N = 8) in the pre- and post-exposure sessions. While the tones were presented to the left ear in the exposure phase in the same-ear condition, the tones were presented to the right ear in the different-ear condition.

## Results

### Experiment 1: Frequency Selectivity

In order to determine the amount of visual displacement that corresponded to PSS, we estimated the 50% point of rightward motion perception by fitting a cumulative normal-distribution function to each individual’s data using a maximum likelihood curve fitting technique.

**Figure 2:**
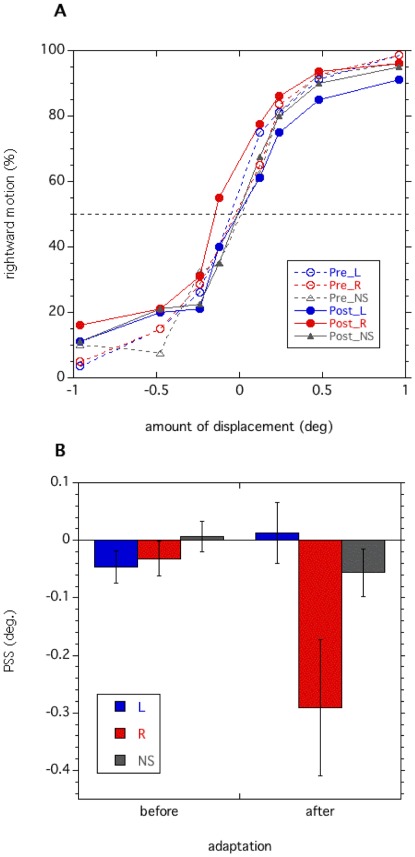
Sound-contingent visual motion aftereffect. The proportion of rightward motion perception of visual stimuli as a function of the degree of physical displacement of visual stimuli. Positive values indicate physical rightward visual motion on the horizontal axis and negative values indicate physical leftward visual motion. Blue lines represent the results for the leftward sound condition, red lines represent the rightward sound condition, and the black line the no-sound condition. While lined symbols represent the results obtained prior to the adaptation period, filled ones represent the results after the adaptation. The dashed horizontal line indicates 50% point of rightward motion perception (the point of subjective stationarity: PSS). (B) Estimated PSSs. Positive values represent the shift of PSS in the direction of the leftward visual motion. Blue bars represent the results for the leftward sound condition; red bars, the rightward sound condition; and grey bars, no sound condition. The error bar denotes the standard error of the mean.

When the adapter and test tone pairs were consistent (400–2100 Hz), the sounds did not affect the perception of visual motion before exposure to an apparent motion (Figure 2). After the exposure, however, the sounds acquired driving effects for visual motion. While the PSS shifted in the direction of the leftward visual motion in the rightward sound condition, it shifted in the direction of the rightward visual motion in the leftward sound condition. There was an asymmetry for the aftereffect: the aftereffect is stronger in the rightward direction than in the leftward. This asymmetry was also found in our previous studies [8], [11], such that a stronger aftereffect was observed for the motion toward the peripheral visual field relative to that toward the foveal one. We have found that motion perception tends to be clearer for the motion toward the peripheral visual field, although we cannot provide a clear explanation for this [8], [11].

A two-way repeated measures analysis of variance (ANOVA) with exposure (2; pre/post) × sound conditions (3; leftward−/rightward−/no-sound) showed main effects from the sound conditions [F(2,14) = 4.12, *p*<0.05]. An interaction between these factors was also significant [F(2,14) = 5.03, *p*<0.05]. With regard to one simple main effect of post-exposure [F(2,28) = 8.78, *p*<0.01], a post hoc test (Tukey’s HSD) found a significant difference in the PSSs of the rightward and the leftward sound conditions [p<0.05] as well as in the PSSs of the rightward and the no sound condition [*p*<0.05]. In contrast, the simple main effect of pre-exposure was not significant [F(2,28) = 0.26, *p* = 0.7]. These results indicate that after prolonged exposure to visual apparent motion accompanied by tones, the tones became drivers for the perception of illusory motion.

Then, we analyzed the effect of test sound frequencies. In line with the previous study [8], the sound-contingent visual motion aftereffect was demonstrated in a positive manner: the sounds induced rightward visual motion perception in the rightward sound conditions. More leftward visual motion was necessary to cancel out this sound-induced illusory visual motion, so that the PSSs shifted to the leftward visual motion. The reverse was true for the leftward sound condition (Figure 2). We also found that the PSSs in the no-sound condition were around 0 deg. of visual motion and did not differ among the exposures. Based on these findings, we calculated the magnitude of the aftereffect as the distance between the PSSs of the leftward or the rightward sound conditions and the ones of the no-sound condition. We first estimated the amount of PSS shift by subtracting the PSSs in the post-exposure from those in the pre-exposure condition in each sound condition. Then, the aftereffect’s magnitude was computed using the following formula:

L-magnitude: PSS shift (No sound)–PSS shift (Leftward sound)

R-magnitude: PSS shift (No sound)–PSS shift (Rightward sound)

**Figure 3:**
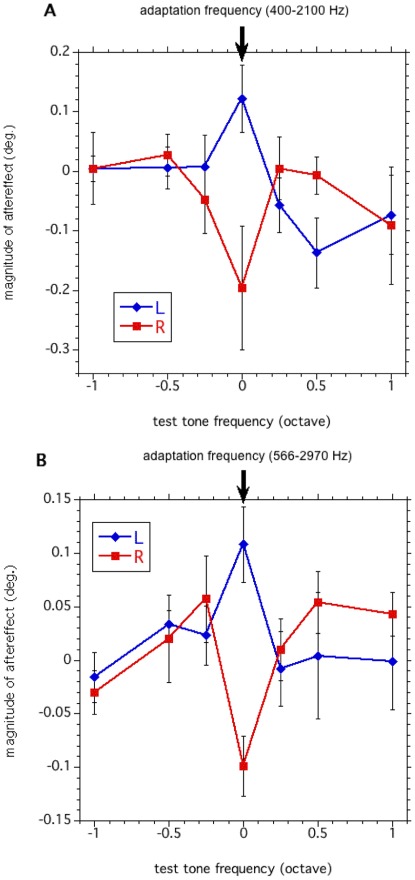
Frequency selectivity of the aftereffect. The magnitude of the aftereffect as a function of test tones in the variable test frequency condition. The magnitude of the aftereffect was calculated subtracting the PSS-shift of each sound condition from the PSS-shift of the NS condition. (A) The right visual field and 400–2100 Hz adaptor combination. (B) The left visual field and 566–2970 Hz adapter combination. For further explanation of how the magnitude was estimated, see text. The mean of the six participants is shown. Error bars indicate s.e. across participants.

Figure 3 presents the magnitude of the aftereffect as a function of test frequencies. The larger values indicate that stronger positive aftereffects occurred. Surprisingly, there was no cross-frequency transfer of the aftereffect. For all test frequencies and all participants, the magnitude of the aftereffect was found to be largest when the frequencies of the adapter and test tone pairs were identical (400–2100 Hz). The aftereffect decreased as the frequency difference between the adapter and test tone pairs increased, and virtually disappeared at a difference of one-half octave. A two-way ANOVA showed that the interaction between the sound and test tone frequency condition is significant [F(6,42) = 3.81, p<0.01]. Post hoc tests (Tukey’s HSD) showed the significant differences in the magnitude of aftereffect between the rightward and leftward sound conditions measured when test tone frequency was presented at 400–2100 Hz, the same as the adapter frequency (p<0.01).

In order to confirm the results of our generalization tests, we analyzed the data using a 566–2970 Hz adapter tone (Figure 3B). Again, with regard to the calculated magnitude of the aftereffect, a two-way ANOVA showed that the interaction between the sound and test tone frequency condition is significant [F(6,42) = 2.85, p<0.05]. Post hoc tests (Tukey’s HSD) showed the significant differences in the magnitude of aftereffect between the rightward and leftward sound conditions measured when the test tone frequency was presented at 566–2970 Hz (p<0.01). These results using another frequency band for the adapter tone confirmed that the sound-contingent visual motion aftereffect is characteristically frequency selective.

When the tone frequencies of the adapter and the test tone pairs were the same, the aftereffect was strong. The results of Experiment 1 showed that the sound-contingent visual motion aftereffect occurs in a frequency-selective manner.

### Experiment 2: Ear Selectivity

In this experiment we tested whether the sound-contingent visual motion aftereffect would transfer in an interaural manner.

**Figure 4:**
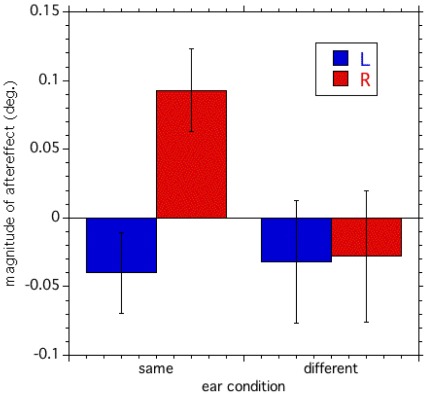
Interaural transfer of the aftereffect. The magnitude of the aftereffect is shown for the “Same” condition, in which both the adapter and test tones were presented to the same (left) ear, and for the “Different” condition, in which the adapter was presented to the right ear and test tones to the left ear. The mean of the six participants is shown. Error bars show s.e. across participants.

As in Experiment 1, we calculated the magnitude of the aftereffect for each ear condition (Figure 4). A two-way ANOVA showed that the interaction between the sound and ear condition is significant [F(1,7) = 6.66, p<0.05]. Post hoc tests (Tukey’s HSD) showed the significant differences in the magnitude of aftereffect between the rightward and leftward sound conditions measured when the adapter and the test tone pairs were presented to the same ears. Thus, the sound-contingent visual motion aftereffect is highly selective in the ear.

## Discussion

In the present study, we investigated whether the sound-contingent visual motion aftereffect is specific to the adapted frequency and ear. We found that the aftereffect was significantly reduced when the adapter and test tone pairs were presented in different sound frequency regions and to different ears.

Audiovisual cross-modal adaptation effects have been reported. For example, adaptation to a constant temporal lag between audio and visual stimuli can bias subsequent temporal order judgment [12]–[15]. It has further been shown that the temporal lag adaptation effect can transfer across different ears. Audiovisual cross-modal adaptation effects have also been reported for ventriloquism aftereffects [16]–[20]. The apparent location of test sounds shifts in the direction of the previously presented visual stimulus following prolonged exposure to auditory and visual stimuli presented in inconsistent locations (e.g. [20]). Substantial ventriloquism aftereffects have been observed across a four-octave range of sound frequencies [16], [17]. While frequency-specific adaptation effects have also been reported [19], [20], the frequency range was quite wide, such as more than 2 octaves. In the present study, we systematically manipulated the sound frequency within a 1-octave range and showed that the aftereffect disappeared with a difference of one-fourth octave. Our findings provide pioneering evidence for the existence of a high selectivity of sound frequency in auditory processing in the audiovisual aftereffect. These results alleviate the concern that sound-contingent visual motion aftereffects might simply be due to a response bias in the decision-making process.

We found robust ear selectivity of the aftereffect. However, the magnitude of aftereffect with the monaural adaptation (Experiment 2) was weaker than that with the binaural adaptation (Experiment 1). One might assume that the dichotic presentation of sounds would be effective on the aftereffect and would involve higher brain areas. However, the S:N ratio of auditory input was improved in the dichotic presentation compared to the monaural presentation [21]. Therefore, the auditory input of low S:N ratio might attenuate the magnitude of the aftereffect.

The present finding demonstrates that the sound-contingent visual motion aftereffect is specific to both the adapted frequency and the adapted ear. This result indicates that the auditory processing governing the establishment of novel audiovisual associations is at least somewhat selective. This selectivity might indicate that early-stage auditory mechanisms underlie the aftereffect examined here, but it is also possible that the sound-contingent visual motion aftereffect could be mediated by top-down processes, such as attention. For instance, it has been shown that location-specific adaptation is modulated by attention in the visual modality [22]. Further, the traditional view of low-level location-specific visual perceptual learning was challenged by a recent study showing that learning transferred between eyes when a double-training procedure was used [23]. Further research should clarify the effect of attention processes on the sound-contingent visual motion aftereffect. One possibility is to use indiscriminable sounds, which would prevent participants from attending to a specific audiovisual pair.
